# Analysis of the complete genome sequences of *Clostridium perfringens* strains harbouring the binary enterotoxin BEC gene and comparative genomics of pCP13-like family plasmids

**DOI:** 10.1186/s12864-022-08453-4

**Published:** 2022-03-23

**Authors:** Kengo Ueda, Kazuki Kawahara, Narumi Kimoto, Yusuke Yamaguchi, Kazuhiro Yamada, Hiroya Oki, Takuya Yoshida, Shigeaki Matsuda, Yuki Matsumoto, Daisuke Motooka, Kentaro Kawatsu, Tetsuya Iida, Shota Nakamura, Tadayasu Ohkubo, Shinya Yonogi

**Affiliations:** 1 Laboratory of Biophysical Chemistry, Graduate School of Pharmaceutical Sciences, Osaka University, 1-6 Yamadaoka, Suita, Osaka, 565-0871 Japan; 2 Department of Microbiology and Medical Zoology, Aichi Prefectural Institute of Public Health, 7-6 Nagare, Tsujicho, Kita-ku, Nagoya, Aichi 462-8576 Japan; 3 Department of Infection Metagenomics, Genome Information Research Center, Research Institute for Microbial Diseases (RIMD), Osaka University, 3-1 Yamadaoka, Suita, Osaka, 565-0871 Japan; 4 Department of Bacterial Infection, Research Institute for Microbial Disease (RIMD), Osaka University, 3-1 Yamadaoka, Suita, Osaka, 565-0871 Japan; 5 Division of Microbiology, Osaka Institute of Public Health, 1-3-69 Nakamichi, Higashinari-ku, Osaka, Osaka 537-0025 Japan; 6 Center for Infectious Disease Education and Research (CiDER), Osaka University, 3-1 Yamadaoka, Suita, Osaka, 565-0871 Japan

**Keywords:** *C. perfringens*, Binary enterotoxin, Horizontal gene transfer, Conjugative plasmid, pCP13-like family plasmid

## Abstract

**Background:**

BEC-producing *Clostridium perfringens* is a causative agent of foodborne gastroenteritis. It was first reported in 2014, and since then, several isolates have been identified in Japan and the United Kingdom. The novel binary ADP-ribosylating toxin BEC, which consists of two components (BECa and BECb), is encoded on a plasmid that is similar to pCP13 and harbours a conjugation locus, called Pcp, encoding homologous proteins of the type 4 secretion system. Despite the high *in vitro* conjugation frequency of pCP13, its dissemination and that of related plasmids, including *bec*-harbouring plasmids, in the natural environment have not been characterised. This lack of knowledge has limited our understanding of the genomic epidemiology of *bec*-harbouring *C. perfringens* strains.

**Results:**

In this study, we determined the complete genome sequences of five *bec*-harbouring *C. perfringens* strains isolated from 2009 to 2019. Each isolate contains a ~ 3.36 Mbp chromosome and 1–3 plasmids of either the pCW3-like family, pCP13-like family, or an unknown family, and the *bec*-encoding region in all five isolates was located on a ~ 54 kbp pCP13-like plasmid. Phylogenetic and SNP analyses of these complete genome sequences and the 211 assembled *C. perfringens* genomes in GenBank showed that although these *bec*-harbouring strains were split into two phylogenetic clades, the sequences of the *bec*-encoding plasmids were nearly identical (>99.81%), with a significantly smaller SNP accumulation rate than that of their chromosomes. Given that the Pcp locus is conserved in these pCP13-like plasmids, we propose a mechanism in which the plasmids were disseminated by horizontal gene transfer. Data mining showed that strains carrying pCP13-like family plasmids were unexpectedly common (58/216 strains) and widely disseminated among the various *C. perfringens* clades. Although these plasmids possess a conserved Pcp locus, their ‘accessory regions’ can accommodate a wide variety of genes, including virulence-associated genes, such as *becA*/*becB* and *cbp2*. These results suggest that this family of plasmids can integrate various foreign genes and is transmissible among *C. perfringens* strains.

**Conclusion:**

This study demonstrates the potential significance of pCP13-like plasmids, including *bec*-encoding plasmids, for the characterisation and monitoring of the dissemination of pathogenic *C. perfringens* strains.

**Supplementary Information:**

The online version contains supplementary material available at 10.1186/s12864-022-08453-4.

## Introduction

*Clostridium perfringens* is a gram-positive, spore-forming bacterium that causes a variety of diseases in humans and animals. The pathogenicity of *C. perfringens* is mainly attributed to the toxins produced by the bacterium, and more than 20 types of toxins have been reported [1–6]. Each *C. perfringens* strain produces a distinct but limited number of these toxins, and strains were historically classified into five toxinotypes according to their ability to express four types of toxins: α-toxin (phospholipase C; PLC; *plc*), β-toxin (CPB; *cpb*), ε-toxin (ETX; *etx*), and ι-toxin (ITX; *iap*/*ibp*) [1]. This typing scheme was recently updated by adding two more toxins, *C. perfringens* enterotoxin (CPE; *cpe*) and necrotic enteritis toxin B (NetB; *netB*), which cause human gastrointestinal (GI) disease and necrotic enteritis in poultry, respectively, resulting in a new classification with seven toxinotypes (types A–G) [7–10].

Two of the six typing toxin genes are located on the chromosome; *plc*, which is carried by all *C. perfringens* strains, is always located on the chromosome, while *cpe* is both on the chromosome (chromosomal *cpe*) and on a plasmid (plasmid-borne *cpe*). The other toxin genes, *cpb*, *etx*, *iap*/*ibp*, and *netB*, are located on plasmids [11, 12]. In addition to these plasmid-encoded toxins, it was recently shown that *C. perfringens* strains causing GI diseases harbour virulence plasmids encoding novel toxin genes. For example, recently identified *C. perfringens* toxin genes, such as *tpeL* (encoding the large cytotoxin TpeL) reported in 2007; *netB* reported in 2008; and three toxin genes, *netE*, *netF*, and *netG*, reported in 2015 were all found to reside on conjugative plasmids [13–15]. This suggests that conjugative plasmids play an important role in disseminating virulence genes in this bacterium via horizontal gene transfer (HGT), which further increases the toxin repertoire of *C. perfringens* [16]. In 2014, we also reported a novel plasmid-encoded enterotoxin, binary enterotoxin of *C. perfringens* (BEC/CPILE), in strains that caused food poisoning in two outbreaks in Osaka and Tochigi Prefectures in Japan in 2009 and 2010, respectively [17–19]. BEC consists of an enzymatic component (BECa; *becA*) and a cell-binding component (BECb; *becB*) and is homologous to the binary ADP-ribosylating toxin, ι-toxin, which causes gastroenteritis in cattle and rabbits and is encoded by a conjugative plasmid present in type E strains. In contrast, the two isolates, strain OS1 from the Osaka cases and TS1 from the Tochigi cases, that caused these outbreaks were classified as type A strains [17]. Since most reported type A strains do not possess any toxin-encoding plasmids, they were not known to cause GI diseases in humans until *bec*-harbouring strains were reported [16]. This suggests that these toxin genes may be acquired via HGT.

Two conjugative plasmid families of *C. perfringens*, pCW3 and pCP13, have been reported. The archetypical plasmid of the pCW3 family, pCW3, has a conjugation region that is designated as the ‘transfer of clostridial plasmid’ (*tcp*) locus [20]. This plasmid is also a carrier of clinically relevant toxins and antimicrobial resistance genes. These genes are encoded in the accessory or variable region, which integrates multiple foreign genes [20, 21]. The archetypical plasmid of the pCP13 family, pCP13, was initially suspected to be non-conjugative because of the absence of any conjugation loci [22]. Although the dissemination of this plasmid family in natural environments is unknown, it has been reported that pCP13 is conjugative, with an extremely high transfer frequency of ~10^−1^ transconjugants/donor cells, which is comparable to that of a pCW3-like family plasmid [22]. In this study, a locus encoding conjugation machinery that is homologous to the type 4 secretion system (T4SS) and was named the ‘pCP13 *C. perfringens*’ (Pcp) locus was newly defined [22]. Unlike pCW3-like family plasmids, no toxin or antimicrobial resistance genes have been identified in the accessory region of pCP13-like family plasmids, except for a consensus variant of the β-toxin2 gene (CPB2; *cpb2*) that is associated with intestinal diseases in horses and piglets [23]. Therefore, it is noteworthy that the recent sequencing of plasmids in OS1 and TS1 strains revealed that *becA*/*becB* genes, which have been associated with human GI diseases, are located in the accessory regions of ~54 kbp plasmids containing almost identical *Pcp* loci (98% identity) to that of pCP13 [17, 22].

Since the first report of BEC by us [17], three other groups have detected *bec*-harbouring *C. perfringens* strains, including strain W5052, isolated from the faeces of patients with gastroenteritis and food in Tokyo Prefecture, Japan (in October 1997 and June 2003, respectively) [19, 24]; strain CP653, isolated from the faeces of an outpatient in Hokkaido Prefecture, Japan (June 2019) [25]; and strain IQ3 (alternatively named Q135.2), isolated from the faeces of a healthy child in London, the UK (September 2019) [26]. We also detected three more *bec*-harbouring *C. perfringens* strains, O13–19, A18–256, and A19–1, in Japan. Strain O13–19 was isolated from the faeces of a patient with gastroenteritis due to a non-*C. perfringens* foodborne illness in Osaka Prefecture (June 2013). The other two strains, A18–256 and A19–1, were individually isolated from the faeces of patients with foodborne gastroenteritis caused by *bec*-harbouring *C. perfringens* in October 2018 and from oysters purchased at a local market in January 2019, respectively, both in Aichi Prefecture, Japan. Therefore, at least eight isolates with different geographical and temporal origins and variation in the symptomatology of the host have been identified (Table S1). As of 19 December 2020, a total of 211 assembled genomes of *C. perfringens* were deposited in the public database; however, only one (Q135.2) was a *bec*-harbouring strain. Thus, to understand the genomic epidemiology of *bec*-harbouring *C. perfringens* strains, high-throughput sequencing of other *bec*-harbouring *C. perfringens* strains is required.

In this study, we sequenced the genomes of five *bec*-harbouring strains, OS1, TS1, O13–19, A18–256, and A19–1, using a hybrid approach [17]. The obtained genomic information showed that the *bec* genes are encoded on the pCP13-like family plasmids, most of which could have been acquired via HGT events mediated by the Pcp conjugation locus. Comparative genomics of these newly determined five *C. perfringens* genomes along with the 211 assembled genomes in the public database showed that the pCP13-like family plasmids are unexpectedly widespread and are distributed across several *C. perfringens* phylogenetic clades. These findings not only illuminate the mechanisms underlying the dissemination of *bec*-harbouring strains but also shed new light on pCP13-like family plasmids to improve our understanding of *C. perfringens* virulence.

## Results and Discussion

### Complete genome sequencing of *bec*-harbouring *C. perfringens* strains

Five *bec*-harbouring *C. perfringens* isolates, OS1, TS1, O13–19, A18–256, and A19–1, were sequenced using short- and long-read sequencing platforms. This hybrid sequencing approach enabled the raw reads to be assembled into a complete genome. The coverage of the short reads was >45× and that of the long reads was >140×, which is sufficient to obtain complete genome sequences with high accuracy (Tables 1 and S2–1-3). These genomes were confirmed to be *C. perfringens* at the species level based on the average nucleotide identity (ANI) and digital DNA-DNA hybridisation (dDDH) values of ≥97.1% and ≥ 74.1%, respectively (Table 1). These genomes had 28.30–28.43% GC content and contained an average of 3023 CDS, 96 tRNA genes, and 31 rRNA genes (Table 1).

**Table 1:** General features and statistics of the *bec*-harbouring *C. perfringens* genomes

	OS1	TS1	O13–19	A18–256	A19–1
Long read platform	PacBio	PacBio	MinION	MinION	MinION
Short read platform	MiSeq	MiSeq	MiSeq	MiSeq	MiSeq
No. of sequences	4	3	3	3	2
Genome size (bp)	3,367,796	3,508,738	3,459,661	3,520,477	3,405,579
GC (%)	28.43	28.32	28.30	28.32	28.36
CDS	2956	3077	3013	3075	2997
tRNAs	96	97	95	96	94
rRNAs	33	30	30	30	30
ANI (%)	98.2	97.1	97.1	97.1	97.1
dDDH (%)	85.0	74.4	74.1	74.6	75.4

**Fig. 1:**
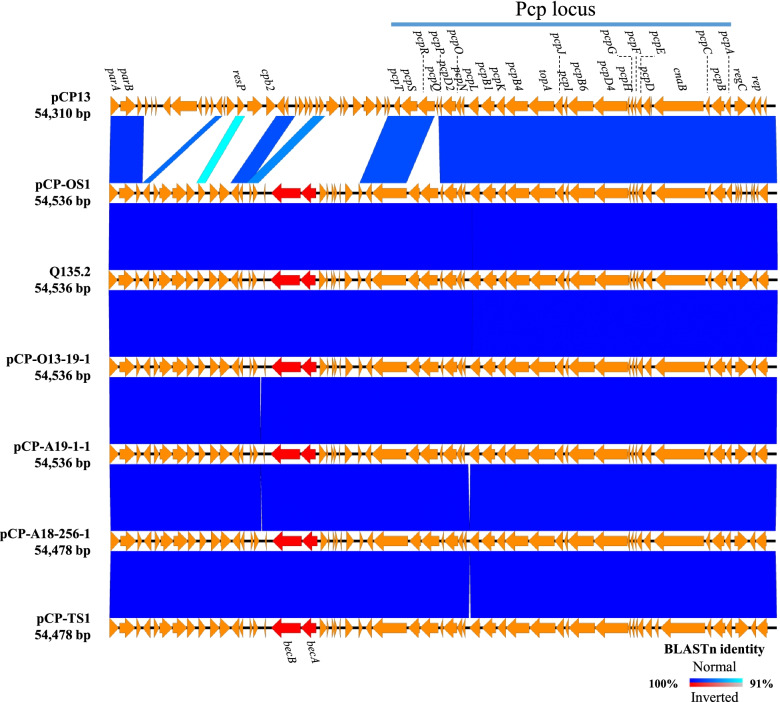
Sequence comparison map of the reference plasmid pCP13 and five *bec*-encoding plasmids. The *bec*-encoding plasmid of strain Q135.2 is labelled with its strain name. The *becA* and *becB* genes are shown as red arrows, and the other genes are shown as orange arrows. The region encoding the highly conserved Pcp locus is indicated as a light blue bar at the top of the figure

Analysis of the complete genome sequences unambiguously showed that each strain had a chromosome with an average length of 3.36 Mbp and one to three plasmids with distinct sizes (range, 14,826–54,530 bp; Table 2). All five isolates harboured an ~54 kbp plasmid (pCP-OS1, pCP-TS1, pCP-O13–19-1, pCP-A18–256-1, or pCP-A19–1-1) containing *becA*/*becB* genes with identical nucleotide sequences (Fig. 1 and Table 2). In addition, each of these five *bec*-encoding plasmids possesses a highly conserved Pcp locus (>97% nt identity), which is characteristic of pCP13-like family conjugative plasmids and makes these five *bec*-encoding plasmids members of the pCP13-like family (Fig. 1 and Table S3).

**Table 2:** Sizes of the chromosomes and plasmids in the *bec*-harbouring *C. perfringens* strains

Strain	Chromosome	Plasmids			
	size (bp)	***bec***-encoding	Size (bp)	Other	Size (bp)
OS1	3,256,834	pCP-OS1	54,536	pCP-OS1–2	41,600
				pCP-OS1–3	14,826
TS1	3,418,761	pCP-TS1	54,478	pCP-TS1–2	35,499
O13–19	3,357,727	pCP-O13–19-1	54,536	pCP-O13–19-2	47,265
A18–256	3,418,734	pCP-A18–256-1	54,478	pCP-A18–256-2	47,265
A19–1	3,350,958	pCP-A19–1-1	54,536	–	–

Four of the isolates (except A19–1) had one or two other plasmids that were distinct from the *bec*-encoding plasmid (Table 2). The O13–19 and A18–256 isolates had ~47 kbp plasmids, which were designated as pCP-O13–19-2 and pCP-A18–256-2, respectively, of identical length and high sequence similarity (99.64% nt identity). These plasmids were also nearly identical (99.39 and 99.38% nt identity for pCP-O13–19-2 and pCP-A18–256-2, respectively) to the conjugative plasmid pCW3, which is the archetypal tetracycline-resistant plasmid encoding the *tetA(P)* and *tetB(P)* genes (Fig. S1 and Table S3) [20]. Thus, we confirmed the coexistence of pCP13-like and pCW3-like family plasmids in a single *C. perfringens* strain. The remaining three plasmids, pCP-OS1–2 and pCP-OS1–3 in strain OS1 and pCP-TS1–2 in strain TS1 did not have meaningful homology with any previously reported *C. perfringens* plasmid.

### Exploring the clostridial toxin and antimicrobial resistance genes, CRISPR regions, and prophage regions in the *C. perfringens* strains

**Fig. 2:**
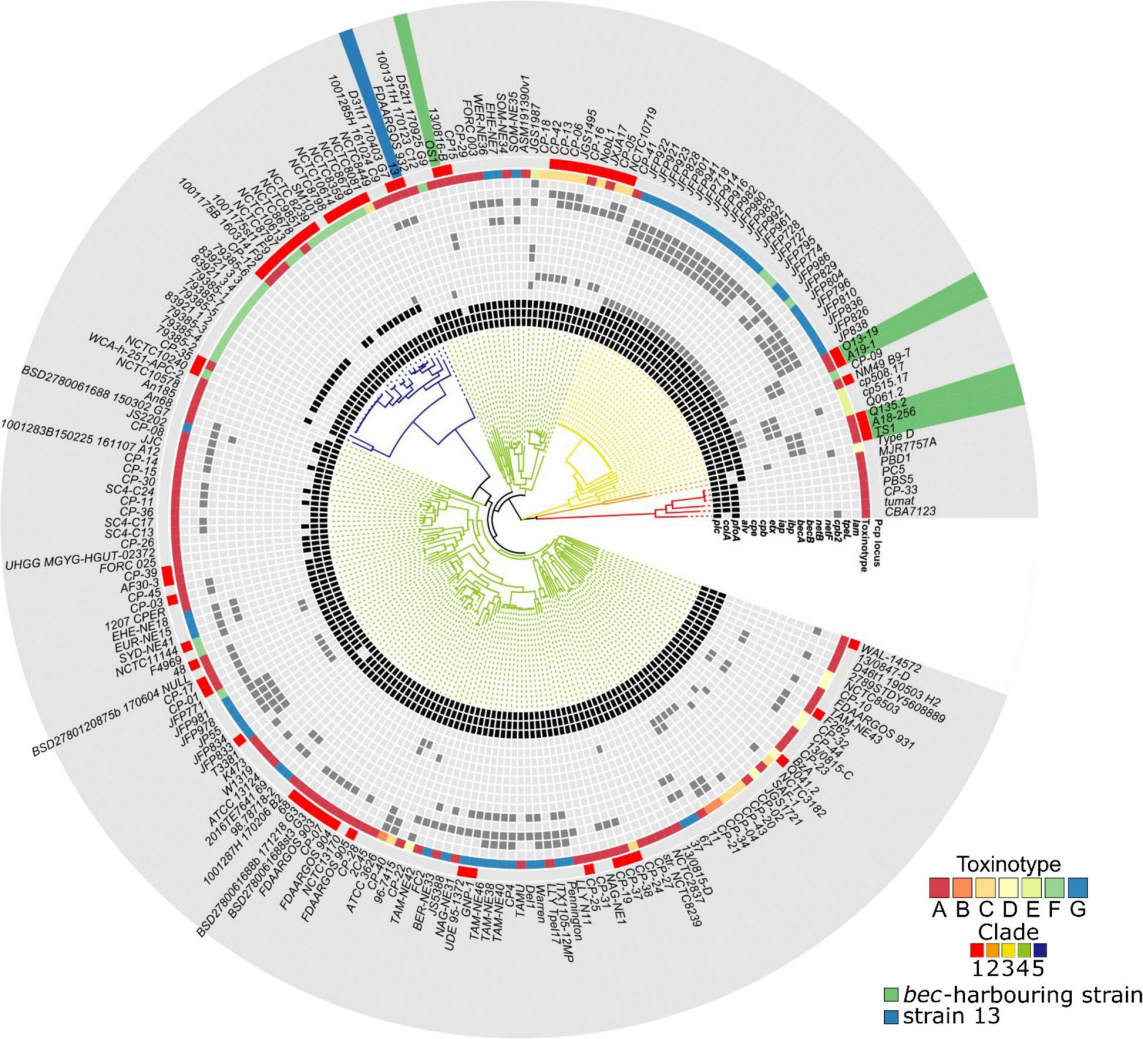
Maximum likelihood phylogenetic tree of 216 *C. perfringens* strains. According to their phylogenetic relationships, the genomes were classified into five clades. The presence and absence of typical *C. perfringens* toxin genes, such as chromosomal *plc*, *colA*, *pfoA*, *cpb2*, and *cpe* (chromosomal or plasmid-borne), and plasmid-borne *netB*, *alv*, *netF*, *tpeL*, *cpb*, *etx*, *becA*, *becB*, *iap*, *ibp*, and *lam* (encoding the thermolysin-like metalloprotease called lambda toxin) [30], are indicated as different colour cells: black for the presence of chromosomal toxin genes, grey for the presence of plasmid-borne toxin genes, and light grey for the absence of the toxin gene. The toxinotyping of *C. perfringens* was determined based on the classification scheme proposed by Rood and co-workers and is indicated with seven colours [10]. In this study, the strains harbouring *netB* and *cpe* were classified as type G. The presence of the Pcp locus is shown as a red cell. Strain 13, which has pCP13, a representative plasmid of the pCP13-like family, is highlighted in blue, and the six *bec*-harbouring strains are highlighted in green

We explored the clostridial toxin genes, antimicrobial resistance genes, CRISPR regions, and prophage regions in the five complete genomes of the sequenced *bec*-harbouring strains. Of the six typing genes, *plc* was solely detected on the chromosome, and five strains were therefore classified as type A [27]. The non-typing toxin genes, collagenase gene (*colA*), perfringolysin O gene (*pfoA*), alpha-clostripain gene (*cloSI*), five hyaluronidase genes (*nagH*, *nagI*, *nagJ*, *nagK*, and *nagL*), and three sialidase genes (*nanH*, *nanI*, and *nanJ*), were shared by all five strains and were located on their chromosomes (Fig. 2 and Table S3). Four strains, excluding OS1, had an alveolysin (*alv*) gene on their chromosome. This toxin, belonging to a family of thiol-activated cytolysins, is frequently found in *Bacillus alvei* [28], but is less common in *C. perfringens* strains [29]. It is interesting to note that no known virulence genes were found on either of the plasmids in the *bec*-harbouring strains, except the *becA/B* genes (Fig. 2 and Table S3).

Regarding antimicrobial resistance genes, all five isolates had a tetracycline resistance gene, *tetM*, on their chromosomes. Strain OS1 also contained *tetA(P)* and chloramphenicol resistance protein (*crp*) genes within a putative prophage region on the chromosome. Two strains, O13–19 and A18–256, expectedly possessed two tetracycline resistance genes, *tetA(P)* and *tetB(P)*, on their ~47 kbp pCW3-like plasmids (Fig. S1) [20].

Strains OS1, TS1, O13–19, and A18–256 carried one or two CRISPR regions on their chromosomes, but A19–1 did not (Table S3). All chromosomes and non-*bec*-encoding plasmids, excluding the ~15 kbp plasmid of strain OS1, encoded one to three putative prophage sequences, including ‘incomplete’, ‘questionable’, and ‘intact’ sequences. In contrast, none of the *bec*-encoding plasmids harboured any sequence associated with CRISPR or prophage (Table S3).

### Phylogenetic analysis of *C. perfringens*

To elucidate the phylogeny of the *bec*-harbouring strains, we constructed a maximum likelihood phylogenetic tree based on the core genome of 216 *C. perfringens* strains, including the five newly determined complete genomes and others extracted from GenBank (Fig. 2). The toxinotype and the presence or absence of typical *C. perfringens* toxin genes in each strain are shown in Fig. 2. The phylogenetic tree suggests that *C. perfringens* strains are divided into five major clades, as was reported previously by Feng et al. [31]. Clade 4 consists of a large number of strains that can be further divided into several clades based on hierarchical Bayesian clustering by RhierBAPS, as was also pointed out by Feng et al. [31, 32] (Fig. S2 and Table S4). In the present study, we combined these clades into one large clade to eliminate complexity (Fig. 2).

Of the six *bec*-harbouring isolates, including strain Q135.2 from the UK [26], five (Q135.2, TS1, O13–19, A18–256, and A19–1) were clustered in clade 3 (Fig. 2). Clade 3 mainly consisted of a large collection of ‘JFP’ strains, which were isolated from a North American dog and in a study of horse *C. perfringens* haemorrhagic enteritis [33]. These strains were categorised as either type F or G, and they possess plasmid-borne *cpe* or *netB* and *plc*, respectively. Among the four currently known type E strains, three strains (cp508.17, cp515.17, and Q061.2) were also clustered in this clade. Given that the five *bec*-harbouring strains were categorised as toxinotype A, the phylogenetic analysis showed that clade 3 contained strains, most of which possessed plasmid-borne toxin genes, with relatively diverse toxinotypes (A, E, F, and G; Fig. 2).

While most of the *bec*-harbouring strains belonged to clade 3, strain OS1 was classified into clade 4 along with strain 13 carrying pCP13 [22], which encodes a conjugative Pcp locus and has a 38 kbp region with sequence similarity to *bec*-encoding plasmids [17, 22]. Considering that the *bec*-encoding plasmids carry a conjugative Pcp locus, we hypothesised that the distribution of *bec* genes across different *C. perfringens* clades was caused by an inter-clade HGT event.

### Detection of HGT events among six *bec*-harbouring strains based on analysis of InDels and SNPs

We compared six *bec*-encoding plasmids, including the plasmids sequenced in this study and one (pIQ3b) from strain Q135.2 [26] (Fig. 1), which showed that the sequence similarity among these six *bec*-encoding plasmids was exceptionally high (>99.81%). The plasmid sequences were then aligned using *parA* as a starting point, and slight differences in SNPs and InDels were confirmed (Tables 3 and 4). These differences consequently divided these plasmids into two groups with distinct sequence lengths: group A (54,478 bp; TS1 and A18–256) and group B (54,536 bp; OS1, O13–19, A19–1, and Q135.2). The group A and B plasmids had insertions of 12 nucleotides at position 49,752–49,763 and four insertions of 19, 24, 15, and 12 nucleotides at positions 12,922–12,940, 49,639–49,662, 50,103–50,117, and 53,236–53,247, respectively (Table 3). In each group, several minor differences in SNPs were detected, at position 45,011 in group A and at positions 12,297, 28,267, 46,299, and 50,695 in group B, which may reflect their slightly different genetic traits. These differences were detected in all plasmids except for two, pCP-O13–19-1 and pCP-A19–1-1 (Table 4), as the sequences of these two plasmids were completely identical, indicating that strains O13–19 and A19–1 acquired their plasmids via recent HGT events or vertical inheritance from a common ancestor, although these strains were isolated from temporally and geographically different locations (June 2013 in Osaka and January 2019 in Aichi Prefecture, Japan, respectively) and from different sources, that is, a patient with gastroenteritis and oysters (Table S1).

**Table 3:** Summary of the InDel regions in the *bec*-encoding plasmids

Region	Group A		Group B			
	TS1	A18–256	A19–1	O13–19	Q135.2	OS1
12,922–12,940	–	–	+	+	+	+
49,639–49,662	–	–	+	+	+	+
49,752–49,763	+	+	–	–	–	–
50,103–50,117	–	–	+	+	+	+
53,236–53,247	–	–	+	+	+	+

**Table 4:** Summary of the SNPs in the *bec*-encoding plasmids

Position	Group A		Group B			
	TS1	A18–256	A19–1	O13–19	Q135.2	OS1
582	A	A	G	G	G	G
6503	A	A	G	G	G	G
11,473	T	T	C	C	C	C
12,297	C	C	C	C	C	T
22,191	T	T	G	G	G	G
25,350	A	A	C	C	C	C
25,785	A	A	G	G	G	G
28,267	A	A	A	A	G	A
37,420	A	A	G	G	G	G
37,720	G	G	A	A	A	A
40,721	A	A	G	G	G	G
45,011	A	C	C	C	C	C
46,299	C	C	C	C	C	T
46,478	T	T	C	C	C	C
48,030	T	T	C	C	C	C
48,848	A	A	G	G	G	G
50,035	G	G	T	T	T	T
50,695	C	C	C	C	C	A
50,966	T	T	C	C	C	C
53,229	A	A	G	G	G	G

To further determine whether the *bec*-encoding plasmids were acquired by vertical or horizontal gene transfer, we compared the accumulation of SNPs among all possible pairs of *bec*-encoding plasmids and cognate chromosomes of the *bec*-harbouring *C. perfringens* strains (Tables 5, 6 and S5). In contrast to draft or whole genomes, complete genome information allows us to calculate the numbers and accumulation rates of SNPs for the plasmids and chromosomes separately, even if a strain contains more than one type of plasmid.

**Table 5:** Chromosomal pair-wise SNPs rates (%) among *bec*-harbouring strains

	OS1	TS1	O13–19	A18–256	A19–1	Q135.2
OS1						
TS1	2.43					
O13–19	2.46	1.16				
A18–256	2.43	0.00192	1.16			
A19–1	2.40	1.16	1.08	1.16		
Q135.2	2.39	1.22	1.07	1.22	1.14

**Table 6:** Plasmid pair-wise SNPs rates (%) among the *bec*-harbouring strains

	OS1	TS1	O13–19	A18–256	A19–1	Q135.2
OS1						
TS1	0.0349					
O13–19	0.00551	0.0294				
A18–256	0.0330	0.00184	0.0275			
A19–1	0.00551	0.0294	0	0.0275		
Q135.2	0.00734	0.0312	0.00184	0.0294	0.00184	

The chromosomal pair-wise comparisons of SNPs showed the largest number between strain OS1 and the other strains (64,905–66,800 per 2,710,984 bp, accounting for 2.39–2.46% of the aligned sequences) among all examined pairs (Tables 5 and S5–1), which is consistent with the phylogenetic tree, in which strain OS1 was classified into a distinct clade (clade 4, Fig. 2). These accumulation rates were much higher than those of the plasmid pair-wise SNPs (0.00551–0.0349%; Tables 6 and S5), which supported an inter-clade HGT event between strain OS1 and the other *bec*-harbouring strains. The number of chromosomal pair-wise SNPs detected in the comparisons among the other five strains (except between strains TS1 and A18–256), which were classified into clade 3, were approximately half (28,922–33,047, accounting for 1.07–1.22% of the aligned sequence) of the numbers for strain OS1 (Tables 5 and S5–1). These accumulation rates were also much higher than those of the plasmid pair-wise SNPs (0–0.0312%; Tables 6 and S5). The number of chromosomal pair-wise SNPs between strains TS1 and A18–256 was remarkably small (52, accounting for 0.00192% of the aligned sequence; Tables 5 and S5–1), which strongly suggested that these two strains, isolated from two distinct outbreaks, originated from nearly clonal strains.

The accumulation rates of SNPs were statistically analysed. The rate of plasmid pair-wise SNPs between strains TS1 and A18–256 was not notably different from that of the chromosomal SNPs (*p* = 1, z = −0.04), which indicates that the *bec*-encoding plasmid was vertically inherited between these two strains. In contrast, the accumulation rates of the other plasmid pair-wise SNPs were much lower than those of the chromosomes (*p* < 2.2e-16, −37.01 ≤ z ≤ −24.19; Table S6). Consequently, these statistical results further supported the conclusion that the *bec*-encoding plasmids were inherited not only via inter-clade but also via intra-clade HGT.

### Distribution of pCP13-like family plasmids

Our comparative analysis of complete genome information demonstrated that *bec*-encoding plasmids carry a conjugative Pcp locus, which is characteristic of pCP13-like family plasmids. The pCW3-like family is another family of conjugative *C. perfringens* plasmids that carry the *tcp* locus, and these plasmids are known to be disseminated among *C. perfringens* clades [20, 34]. In contrast, the distribution of pCP13-like family plasmids has not been investigated, and only a limited number of pCP13-like family member plasmids, including pCP13 from strain 13, pCPNY83906550–1 from strain NY83906550, pCPT1 from strain T1, and p1 from strain JXJA17 (Table S7) have been previously known [22, 24, 27, 35].

Thus, we examined the 216 assembled *C. perfringens* genomes, including our five *bec-*harbouring strains, to explore the distribution of pCP13-like family plasmids and found 58 strains (26.85%) likely had a Pcp locus (Fig. 2). Fifty-seven of the strains (the exception being strain NCTC8797, which lacks several parts of the Pcp locus) were confirmed to have both *pcpB4* and *pcpD4* genes, which encode a conjugation-specific VirB4-like ATPase and a VirD4-like coupling protein, respectively, which are crucial for conjugative transfer [22, 36]. The plasmids are distributed broadly across several *C. perfringens* clades (clades 3, 4 and 5), suggesting that a wide variety of *C. perfringens* strains can acquire pCP13-like family plasmids via HGT events.

### Comparative genomics of pCP13-like family plasmids

**Fig. 3:**
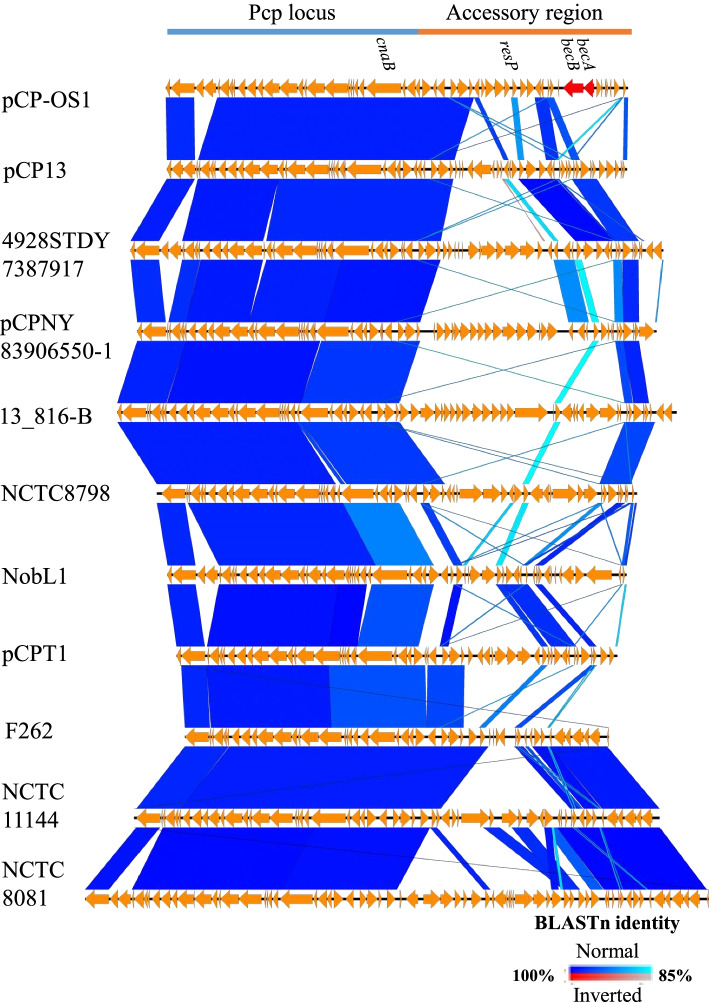
Sequence comparison map of the pCP13-like family plasmids from various *C. perfringens* clades. Seven unnamed plasmid sequences, excluding pCP-OS1, pCP13, pCPNY83906550–1 and pCPT1, are labelled with their carrier strain names. The Pcp locus and accessory region are indicated as light blue and orange bars, respectively, at the top of the figure

To compare the gene structure and organisation of the Pcp locus and accessory regions of the pCP13-like family plasmids, we selected 11 plasmids as representatives from various *C. perfringens* clades that contain Pcp locus-harbouring strains based on their sequence alignment (Fig. S3). The results showed that the 11 plasmids shared a highly conserved sequence in the Pcp locus (>92% nt identity) (Fig. 3). Although the conjugation of pCP13-like family plasmids has recently been experimentally shown between two strain 13 derivatives, in future studies, it might be interesting to perform additional conjugation experiments using the strains described above to examine inter-clade transfer of pCP13-like family plasmids.

In the accessory regions, which encode several recombinase genes, the sequence lengths and numbers of encoded genes varied depending on the plasmid (Fig. 3 and Table 7), indicating that this region can incorporate a wide variety of foreign genes, as is also the case for the *becA*/*becB* and *cpb2* genes. Importantly, numerous genes encoded in these regions are still functionally uncharacterized and are currently annotated as ‘hypothetical’ (Table 7). Therefore, further characterisation and monitoring of the genes in this plasmid family are required.

**Table 7:** Annotation of the CDSs in pCP-OS1

No. of CDS	Position (nt) 1–54,536	Size (bp) (strand)	Identical ORFs of pCP13	Gene name	Annotation/putative function
1	1–753	753(+)	PCP01	*parA*	Type I partitioning system ATPase
2	812–2092	1281(+)	PCP02	*parB*	Type I partitioning system centromere binding protein
3	2208–2570	363(+)	PCP03	–	Hypothetical
4	2730–3308	579(−)	PCP12	–	Hypothetical with SMC_N superfamily domain
5*	3620–4033	414(+)	–	–	Hypothetical
6*	4091–5125	1035(+)	–	–	ABC transporter permease
7*	5151–6326	1176(+)	–	–	ABC transporter permease (*FtsX)*
8*	6323–7006	684(+)	–	–	ABC transporter ATP-binding protein
9	7270–7842	573(+)	PCP15	*resP*	Serine recombinase/resolvase (ResP)
10*	8213–8971	759(+)	–	–	Hypothetical
11*	8986–9861	876(+)	–	–	Hypothetical
12	9926–10,570	645(−)	PCP18	–	Hypothetical
13	10,582–10,896	315(−)	PCP19	–	PadR family transcriptional regulator
14	11,456–11,659	204(+)	PCP24	–	Hypothetical
15	11,733–12,122	390(+)	PCP25	–	Hypothetical
16*	12,618–12,737	120(−)	–	–	Hypothetical
17*	13,223–15,622	2400(−)	–	*becB*	Binary enterotoxin component b
18*	15,641–16,900	1260(−)	–	*becA*	Binary enterotoxin component a
19*	17,193–17,786	594(+)	–	–	Sigma-70 family RNA polymerase sigma factor
20*	17,779–17,913	135(+)	–	–	Hypothetical
21*	18,188–18,376	189(+)	–	–	Hypothetical
22*	18,381–18,599	219(+)	–	–	Hypothetical
23*	18,777–18,884	108(−)	–	–	Hypothetical
24*	19,292–19,894	603(+)	–	–	Recombinase family protein
25*	20,361–20,663	303(+)	–	–	Hypothetical
26	20,920–21,417	498(−)	PCP34	*pcpT*	Hypothetical
27	21,473–24,283	2811(−)	PCP35/36	*pcpS/R*	PcpS: Hypothetical, PcpR: ImmA/IrrE family metallo-endopeptidase
28*	24,416–25,345	930(−)	–	–	Restriction enzyme
29*	25,365–26,855	1491(−)	–	–	Eco571 restriction-modification methylase domain-containing protein
30	27,021–27,239	219(−)	PCP38	*pcpP*	Hypothetical
31	27,257–28,390	1134(−)	PCP39	*pcpD2*	Putative relaxase
32	28,393–28,800	408(−)	PCP40	*pcpO*	N-terminal CopG-like ribbon-helix-helix protein (35% coverage of aa sequence)
33	28,804–29,073	270(−)	PCP41	*pcpN*	Hypothetical
34	29,402–30,265	864(−)	PCP43	*pcpL*	Hypothetical
35	30,443–31,558	1116(−)	PCP44	*pcpB1*	Putative peptidoglycan hydrolase
36	31,691–32,383	693(−)	PCP45	*pcpK*	Hypothetical C-terminal Ntf-like transpeptidase domain protein (6% coverage of aa sequence)
37	32,388–34,286	1899(−)	PCP46	*pcpB4*	VirB4-like ATPase
38	34,304–36,415	2112(−)	PCP47	*topA*	Topoisomerase III
39	36,460–37,104	645(−)	PCP48	*pcpJ*	Hypothetical
40	37,220–37,501	282(−)	PCP49	*pcpI*	Hypothetical
41	37,504–39,633	2130(−)	PCP50	*pcpB6*	VirB6-like
42	39,630–42,371	2742(−)	PCP51	*pcpD4*	VirD4-like coupling protein
43	42,358–42,570	213(−)	PCP52	*pcpH*	Hypothetical
44	42,637–42,873	237(−)	PCP53	*pcpG*	Hypothetical
45	42,937–43,131	195(−)	PCP54	*pcpF*	Hypothetical
46	43,124–43,576	453(−)	PCP55	*pcpE*	Spo0A-homologue
47	43,695–44,291	597(−)	PCP56	*pcpD*	Sortase
48	44,535–48,641	4107(−)	PCP57	*cnaB*	Collagen adhesion protein
49	48,786–49,136	351(−)	PCP58	*pcpC*	PemK toxin (type II toxin-antitoxin system)
50	49,260–50,327	1068(−)	PCP59	*pcpB*	Hypothetical
51	50,373–50,858	486(−)	PCP60	*pcpA*	Helix-turn-helix containing DNA-binding regulatory protein
52	51,171–52,274	1104(+)	PCP61	*regC*	LexA-like transcriptional regulator, similar to RegC from pCW3
53	52,388–52,834	447(−)	PCP62	–	Hypothetical
54	52,863–53,780	918(−)	PCP63	*rep*	replication protein

The CDS of *pcpM* was not annotated (identified) by Prokka and was excluded from this table. Asterisks indicate unique CDSs in pCP-OS1 compared to pCP13

Since pCP13-like family plasmids have long been unrecognised, to the best of our knowledge, no detection methods that efficiently identify such family plasmids have been developed. In this study, our comparative genomics revealed extremely high sequence conservation of the Pcp locus among many pCP13-like family plasmids. This result may afford the design of an efficient, cost-effective PCR-based detection system that is helpful for monitoring and determining the virulence potential of pCP13-like family plasmids distributed worldwide.

## Conclusion

In the present study, we reported five complete genomes of *bec*-harbouring *C. perfringens* strains isolated in 2009–2019. Analysis of the genomes showed that all the *bec*-encoding plasmids were pCP13-like plasmids and were disseminated among *C. perfringens* strains via HGT events, except for strains TS1 and A18–256, which appeared to be clonal. A previous study demonstrated the high transfer efficiency of the conjugative pCP13 plasmid *in vitro* [22], and our comparative genomics and data mining approach suggested that pCP13 and other plasmids in this family encoding conjugative Pcp loci were widely disseminated among *C. perfringens* strains (58/216 strains) in the natural environment. Additionally, the accessory regions of this plasmid family were potentially capable of integrating a wide variety of foreign genes, including virulence genes, such as the *bec* gene. These results demonstrate that care must be taken to monitor this newly characterised virulence plasmid in the surveillance of pathogenic *C. perfringens*.

## Methods

### Bacterial strains and genomic DNA extraction

The five *bec*-harbouring *C. perfringens* strains (OS1, TS1, O13–19, A18–256, and A19–1) sequenced in this study were isolated in Japan. OS1, TS1 [17], and A18–256 were isolated from faecal specimens of patients during distinct *C. perfringens* foodborne outbreaks in 2009, 2010, and 2018, respectively; O13–19 was isolated from a faecal specimen of a patient during a non-*C. perfringens* foodborne outbreak in 2013; and A19–1 was isolated from an oyster in 2019.

All strains were anaerobically cultured in GAM broth (Nissui) at 37 °C, and then harvested by centrifugation at 3000 rpm for 20 min and subsequent collection of the cell pellet. Genomic DNA was extracted from the resuspended cells using the DNeasy PowerSoil Kit (QIAGEN).

### Genome sequencing and assembly

The extracted genomic DNA of the five BEC-producing *C. perfringens* strains was sequenced using short and long sequencing platforms. The genomic DNA from strains OS1 and TS1 was sequenced using the PacBio RS II platform (Pacific Biosciences) and the SMRTbell Template Preparation Kit 1.0 (Pacific Biosciences). The genomic DNA from strains O13–19, A18–256, and A19–1 was sequenced using MinION (Oxford Nanopore Technologies) and the SQK-LSK109 1D ligation library preparation kit (Oxford Nanopore Technologies). MiSeq (Illumina) sequencing was also performed for the five strains using a paired-end strategy (2 × 150 cycles or 2 × 250 cycles) with the KAPA Hyper Plus library preparation kit (KAPA Biosystems). The sequencing reads produced by the PacBio RS II platform were assembled using HGAP v3 software [37]. The partial sequencing reads (a total of 3.3 Mbp × 50 lengths of sequencing reads) produced by MinION were assembled using Flye v2.5 software [38]. Assemblies with reads from MinION were corrected using Minimap2 v2.17 [39], and the assembled sequences of the five strains using reads from MiSeq were corrected using Pilon v1.23 [40].

### Sequence determination of six *bec*-encoding plasmids

The sequences of the five assembled *bec*-encoding plasmids were polished using MiSeq reads and Pilon v1.23. The polished sequences were aligned using MAFFT v7.475 and manually checked, followed by Sanger sequencing of any sequences with putative errors.

The raw sequencing reads of Q135.2 were retrieved from the European Nucleotide Archive (ENA) (project PRJEB33762). The reads were quality trimmed and adapter removed using TrimGalore v0.6.4, with default options, followed by *de novo* assembly using Unicycler v 0.4.8 [41].

### Genome annotation and calculating the ANI and dDDH

All assembled sequences were annotated using Prokka v1.14.5 [42]. The sequence of pCP-OS1 was manually annotated using pCP13 as a reference. The sequences encoding the *dnaA* gene were set as the chromosomal sequences. For all assemblies, the average nucleotide identity (ANI) and digital DNA-DNA hybridisation (dDDH) were calculated using fastANI v1.3 [43] and GGDC v2.1 [44], respectively, which confirmed that all ANI and dDDH values were > 95 and > 70%, respectively.

### Phylogenetic analysis of the core genome

A total of 211 assembled genomes of *C. perfringens* were downloaded from GenBank. Phylogenetic trees of 216 genomes, including our five strains, were constructed by the maximum likelihood method using Parsnp v1.2, with strain 13 as the reference genome, using the –x option to remove potential recent recombinations [45]. To determine the genetic distance, a VCF file of the tree was generated using Gingr [45]. The phylogenetic tree was visualised using the R packages phylogram, ggtree [46], ape, and RColorBrewer along with the presence of toxin genes and strain toxinotypes. Population structure was analysed using RhierBAPS with default parameters to assign the 216 strains to phylogenetic clades [32].

### Exploring the genomes for virulence and antimicrobial resistance genes, CRISPR regions, and prophages

The toxin genes in the genome sequences of the 216 *C. perfringens* strains were identified using Abricate v1.0.1 and a custom toxin gene database with default settings (Table S8–1) [47]. For the newly determined genome sequences of the five *bec*-harbouring strains, antimicrobial resistance genes were identified using a custom antimicrobial resistance gene database (Table S8–2) along with nine other databases (NCBI AMRFinderPlus, CARD, Resfinder, ARG-ANNOT, MEGARES, EcOH, PlasmidFinder, VFDB, and Ecoli_VF) supported in the software [47]. CRISPR regions and prophages were identified using MinCED software (v.0.4.2) and PHAge Search Tool Enhanced Release (PHASTER) [48, 49], respectively.

### Comparing SNP accumulation between chromosomes and *bec*-encoding plasmids

The chromosomal sequences of the five newly sequenced strains, OS1, TS1, O13–19, A18–256, and A19–1, and the whole genome sequence of Q135.2 were aligned using Parsnp v1.2. Based on the generated VCF file, the lengths of the common sequences and the numbers of SNPs were determined. The six *bec*-encoding plasmids were also analysed in the same way as the chromosomes, that is, aligned using MAFFT software v7.475, followed by manual confirmation of SNPs and indels. The aligned common sequences were considered to be the core genomes of these plasmids.

The SNP accumulation rates were calculated as the number of SNPs divided by the length of the common sequences, that is, 2,710,984 bp for the chromosomes, and 54,466 bp for the *bec*-encoding plasmids. Binomial tests were conducted for pairwise accumulation of SNPs between chromosomes and plasmids using R version 4.2.0, based on the rates of each chromosome pair.

### Exploring the pCP13-like plasmids in public NGS data

We explored the Pcp locus in the 211 assembled genomes from GenBank using BLASTn using the Pcp locus sequence of pCP13 as a query. Candidate contigs encoding *pcpB4* and *pcpD4* were examined. The strains possessing these genes were identified as probable pCP13-like family plasmid carriers.

### Comparative genomics of the pCP13-like family plasmids

First, 47 pCP13-like plasmids were set using *pcpT* or *pcpS* genes. Then, the sequence of the Pcp locus in each plasmid was extracted. Next, the Pcp locus sequences were aligned using MAFFT v7.475, and a maximum-likelihood phylogenetic tree was constructed using RAxML v.8.2.12, followed by visualisation using FigTree v1.4.4. Based on these results, 11 representative pCP13-like plasmids encoding probable whole sequences were annotated using Prokka v1.14.6 [42] with the default setting and visualised using Easyfig v2.2.2 [50].

## Supplementary Information

**Additional file 1.**

## Data Availability

The sequence data supporting the conclusions of this article are available at DDBJ (accession numbers OS1: AP024969–AP024972, TS1: AP024973–AP024975, O13–19: AP024976–AP024978, A18–256: AP024979–AP024981, A19–1: AP024982 and AP024983).

## Abbreviations

nt: Nucleotide
CRISPR: Clustered Regularly Interspaced Short Palindromic Repeats
SNP: Single Nucleotide Polymorphism
CDS: Coding Sequences
ORF: Open Reading Frame
