# Supplementary material for: Analysis of the complete genome sequences of Clostridium perfringens strains harbouring the binary enterotoxin BEC gene and comparative genomics of pCP13-like family plasmids
Source: BMC Genomics. 2022 Mar 23;23:226. doi: 10.1186/s12864-022-08453-4 (PMC8941779; doi:10.1186/s12864-022-08453-4)
Supplement: Supplementary file 1 — Additional file 1. [file 12864_2022_8453_MOESM1_ESM.pdf]

Supplementary Materials

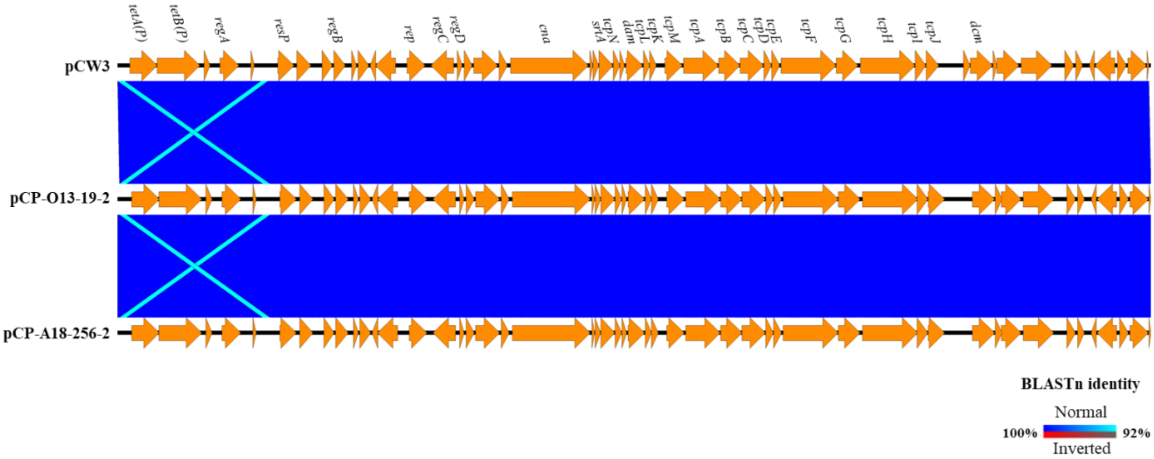

Fig. S1 Plasmid sequence identity comparison map of pCW3 and the pCW3-like family plasmids of strains O13-19 and A18-256. All three plasmid have the tetracycline resistance genes *tetA(P)* and *tetB(P)*.

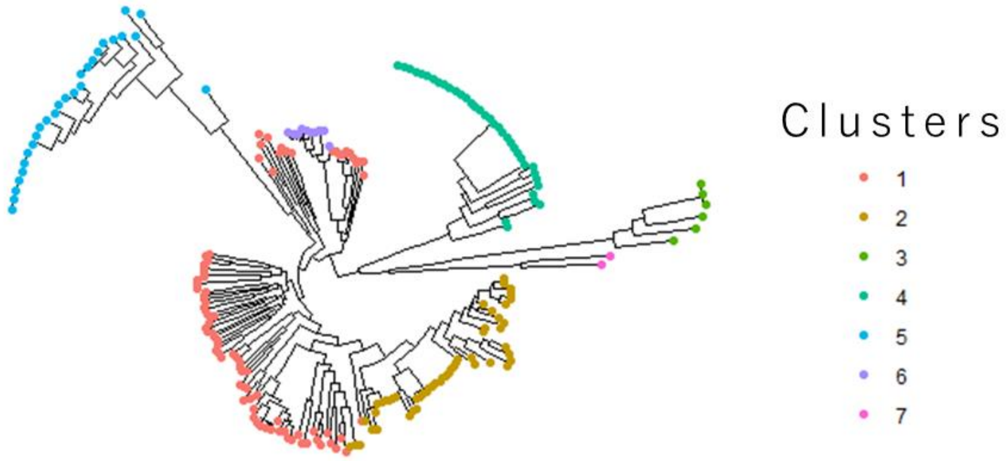

Fig. S2 Phylogenetic tree with the population clusters identified using RhierBAPS [31]. The seven top-level clusters are coloured differently, as shown in the right panel.

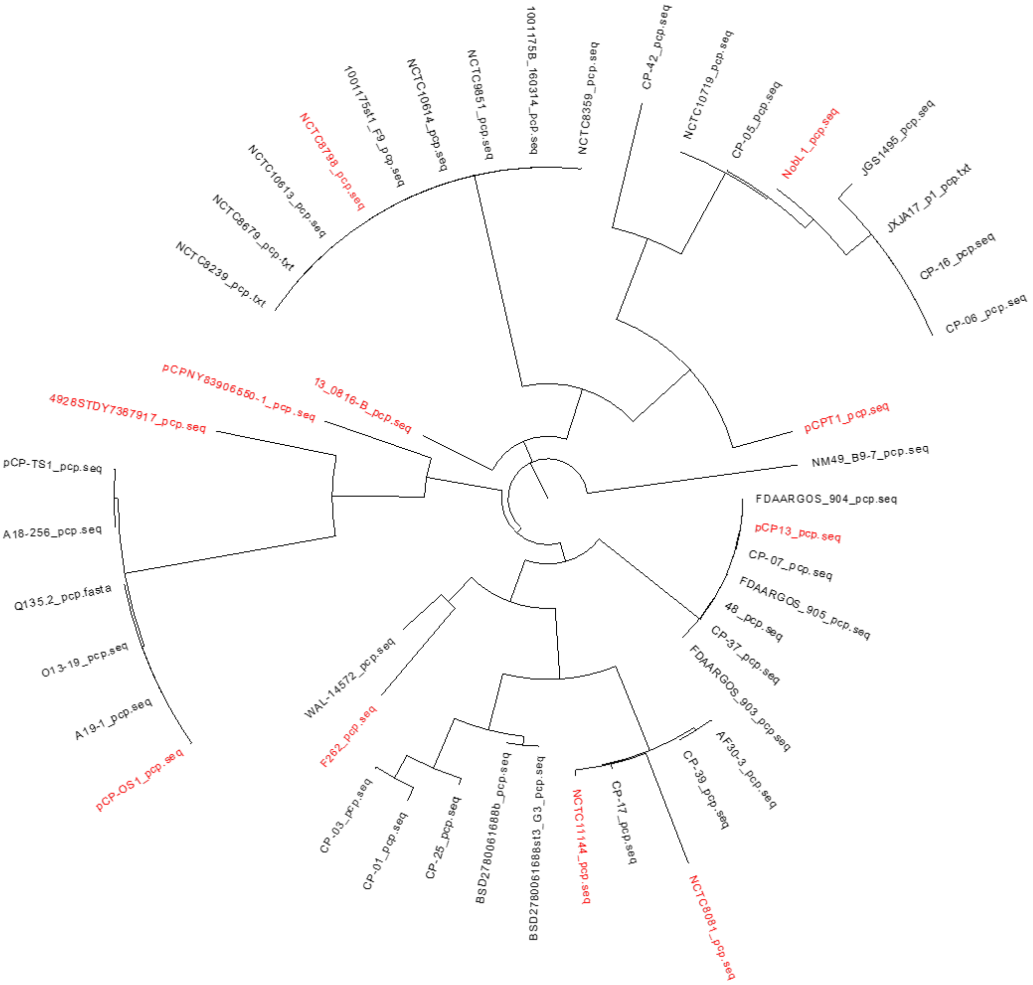

Fig. S3 Phylogenetic analysis of pCP13-like family plasmids. The 11 plasmids, which were selected as representatives of a variety of *C. perfringens* clades, are shown in red.

Table S1 Profiles of BEC-positive strains.

Strain	Year of isolation	Geographic location	Specimen	References
W5052	1997	Tokyo, Japan	Human faeces*	Monma et al. 2015
Unnamed	2003	Tokyo, Japan	Human faeces*	Monma et al. 2015
OS1	2009	Osaka, Japan	Human faeces*	Yonogi et al. 2014
TS1	2010	Tochigi, Japan	Human faeces*	Yonogi et al. 2014
O13-19	2013	Osaka, Japan	Human faeces	This study
A18-256	2018	Aichi, Japan	Human faeces*	This study
A19-1	2019	Aichi, Japan	Oyster	This study
CP653	2019	Hokkaido, Japan	Human faeces	Matsuda et al. 2019
Q135.2	2019	The UK	Human faeces	Kiu et al. 2019

*From a patient of each foodborne outbreak caused by BEC-producing *C. perfringens*.

Table S2-1. Short read sequencing data analysed by MiSeq.

	OS1	TS1	O13-19	A18-256	A19-1
Read length (bp)	251	251	151	151	151
Reads (bp)	154,826,338	174,103,640	361,021,672	384,742,866	464,990,910
Coverage (X)	46	50	104	109	137

Table S2-2. Long read sequencing data analysed by MinION.

	O13-19	A18-256	A19-1
Total reads	804,218	1,149,881	658,924
Total length	2,730,537,575	3,853,040,818	2,709,196,587
Coverage	789	1,095	796
Mean	3,395	3,350	4,111
Min	42	46	43
Median	2,507	2,425	3,228
Max	33,570	32,103	34,515
Best quality	20	21	21
Mean quality	15	14	15
Lowest quality	2	1	2
Longest read quality	12	15	17
N10	10,240	10,024	11,476
N25	8,152	8,059	9,313
N50	5,989	6,009	7,083
N75	3,770	3,873	4,724
N90	1,917	1,897	2,411

Table S2-3. Long read sequencing data analysed by PacBio RS II platform.

	OS1	TS1
Total reads	117,190	227,285
Total length	475,879,292	612,387,193
Coverage	141	175
Mean	4,061	2,694
Min	35	35
Max	47,848	62,049
N50	5,215	3,211

Table S3 Genome descriptions and gene presence in 5 *bec*-harbouring strains.

Strain/ sequences	Plasmid family	GC contents (%)	No. of CDS	Virulence genes	Antimicrobial resistance genes	No. of CRISPR regions	No. of prophages
OS1/		28.43	2,959				
Chromosome		28.50	2,834	*colA*, *pfoA*, *plc*, *cloSI*, *nagH-L*, *nanH-J*	*tetA(P)*, *tetM*	1	2 (1 incomplete, 1 questionable)
pCP-OS1	pCP13-like	25.04	54	*becA*, *becB*	ND	0	0
pCP-OS1-2	NT	28.18	56	ND	*crp*	0	1 (incomplete)
pCP-OS1-3	NT	26.04	15	ND	ND	0	0
TS1/		28.32	3,084				
Chromosome		28.38	2,973	*alv*, *colA*, *pfoA*, *plc*, *cloSI*, *nagH-L*, *nanH-J*	*tetM*	1	3 (1 intact, 1 incomplete, 1 questionable)
pCP-TS1	pCP13-like	25.02	55	*becA*, *becB*	ND	0	0
pCP-TS1-2	NT	27.40	56	ND	ND	0	1 (intact)
O13-19/		28.30	3,013				
Chromosome		28.36	2,909	*alv*, *colA*, *pfoA*, *plc*, *cloSI*, *nagH-L*, *nanH-J*	*tetM*	2	2 (incomplete)
pCP-O13-19-1	pCP13-like	25.04	54	*becA*, *becB*	ND	0	0
pCP-O13-19-2	pCW3-like	27.56	50	ND	*tetA(P)*, *tetB(P)*	0	1 (incomplete)
A18-256/		28.32	3,076				
Chromosome		28.38	2,972	*alv*, *colA*, *pfoA*, *plc*, *cloSI*, *nagH-L*, *nanH-J*	*tetM*	1	3 (1 intact, 1 incomplete, 1 questionable)
pCP-A18-256-1	pCP13-like	25.02	54	*becA*, *becB*	ND	0	0
pCP-A18-256-2	pCW3-like	27.57	50	ND	*tetA(P)*, *tetB(P)*	0	1 (incomplete)
A19-1/		28.36	2,998				
Chromosome		28.41	2,944	*alv*, *colA*, *pfoA*, *plc*, *cloSI*, *nagH-L*, *nanH-J*	*tetM*	0	2 (1 intact, 1 incomplete)
pCP-A19-1-1	pCP13-like	25.04	54	*becA*, *becB*	ND	0	0

NT : not typable, ND : not detected, *crp* : chloramphenicol resistance protein

Table S4 The population clusters of 216 *C. perfringens* strains identified using RhierBAPS.

Isolate	Cluster	Isolate	Cluster
13	1	ATCC_3626	2
CP-14	1	96-7415	2
D31t1_170403_G7	1	TAM-NE38	2
JJC	1	UDE_95-1372	2
CP-02	1	K473	2
SC4-C17	1	JS5388	2
1001285H_161024_C9	1	TAM-NE40	2
11	1	CP4	2
BSD2780061688_150302_G7	1	Warren	2
CP-32	1	BSD2780061688st3_G3	2
OS1	1	CP-22	2
CP-43	1	GNP-1	2
CP-19	1	SYD-NE41	2
CP-20	1	TAMU	2
SC4-C24	1	ITX1105-12MP	2
13/0847-D	1	BSD2780061688b_171218_G3	2
FDAARGOS_931	1	98.78718-2	2
UHGG_MGYG-HGUT-02372	1	W1319	2
NCTC11144	1	68	2
13/0815-C	1	JP55	2
48	1	JFP981	2
SOM-NE35	1	Pennington	2
SAF-1	1	LLY_N11	2
BzA	1	BER-NE33	2
AF30-3	1	NAG-NE31	2
SOM-NE34	1	JFP834	2
FORC_003	1	CP-40	2
CP-24	1	CBA7123	3
CP-08	1	PBS5	3
1207_CPER	1	PBD1	3
str._NCTC8239	1	tumat	3
CP-36	1	CP-33	3
An185	1	PC5	3
CP-29	1	JFP916	4

CP-26	1	JFP804	4
EHE-NE7	1	JP838	4
CP-23	1	JFP983	4
CP15	1	JFP795	4
37	1	A19-1	4
CP-27	1	JFP921	4
NCTC2837	1	JFP801	4
13/0816-B	1	Q061.2	4
2789STDY5608889	1	JFP727	4
WAL-14572	1	O13-19	4
1001283B150225_161107_A12	1	CP-09	4
ASM191390v1	1	JFP941	4
JGS1721	1	JFP986	4
TAM-NE43	1	cp508.17	4
WCA-h-251-APC-2	1	JFP829	4
13/0815-D	1	Q135.2	4
CP-04	1	cp515.17	4
67	1	JFP810	4
CP-17	1	JFP828	4
SC4-C13	1	JFP961	4
CP-37	1	JFP728	4
NCTC10578	1	JFP718	4
CP-21	1	JFP923	4
D46t1_190503_H2	1	JFP992	4
JGS1987	1	JFP836	4
D52t1_170925_G9	1	JFP922	4
JS2202	1	TS1	4
CP-39	1	JFP826	4
CP-45	1	JFP796	4
CP-34	1	JFP914	4
BSD2780120875b_170604_NULL	1	JFP980	4
NAG-NE1	1	A18-256	4
CP-15	1	NM49_B9-7	4
FDAARGOS_932	1	JFP982	4
CP-41	1	JFP774	4
F4969	1	NCTC8081	5

FORC_025	1	NCTC10613	5
NCTC8503	1	NCTC8798	5
1001311H_170123_C12	1	NCTC9851	5
WER-NE36	1	NCTC8359	5
CP-10	1	NCTC_8239	5
F262	1	NCTC8679	5
CP-03	1	79385-4	5
CP-11	1	SM101	5
CP-30	1	79385-2	5
Q041.2	1	79385-1	5
An68	1	83921_3_4	5
NCTC3182	1	83921_3_3	5
CP-38	1	CP-35	5
CP-44	1	79385-7	5
FC2	2	1001175st1_F9	5
T3381	2	79385-6	5
CP-25	2	79385-3	5
2016TE7641_69	2	NCTC8797	5
ATCC_13124	2	1001175B_160314_F9	5
CP-31	2	83921_1_2	5
CP-07	2	NCTC10614	5
TAM-NE42	2	CP-12	5
JFP978	2	NCTC8449	5
1001287H_170206_B2	2	79385-5	5
NCTC13170	2	NCTC8678	5
JFP771	2	NCTC10240	5
CP-28	2	JXJA17	6
TAM-NE46	2	NCTC10719	6
CP-01	2	NobL1	6
EHE-NE18	2	CP-05	6
FDAARGOS_903	2	CP-13	6
2C45	2	CP-16	6
EUR-NE15	2	CP-42	6
FDAARGOS_904	2	CP-06	6
Del1	2	CP-18	6
LLY_Tpel17	2	JGS1495	6

FDAARGOS_905	2	Type_D	7
JFP833	2	MJR7757A	7

Table S5-1 The number of chromosomal pair-wise SNPs from 2,710,984 bp of aligned chromosomal sequence

	OS1	TS1	O13-19	A18-256	A19-1	Q135.2
OS1						
TS1	65925					
O13-19	66800	31561				
A18-256	65944	52	31577			
A19-1	65102	31411	29271	31429		
Q135.2	64905	33029	28922	33047	30900	

Table S5-2 The number of plasmid pair-wise SNPs from the 54,466 bp of aligned plasmid sequence

	OS1	TS1	O13-19	A18-256	A19-1	Q135.2
OS1						
TS1	19					
O13-19	3	16				
A18-256	18	1	15			
A19-1	3	16	0	15		
Q135.2	4	17	1	16	1	

Table S6 Z-scores and p-values comparing plasmid SNPs rates with chromosomal SNPs rates

Strains		Z-score	p-value
OS1	TS1	-36.32	<2.2e-16
OS1	O13-19	-37.01	<2.2e-16
OS1	A18-256	-36.35	<2.2e-16
OS1	A19-1	-36.52	<2.2e-16
OS1	Q135.2	-36.44	<2.2e-16
TS1	O13-19	-24.69	<2.2e-16
TS1	A18-256	-0.04	1
TS1	A19-1	-24.63	<2.2e-16
TS1	Q135.2	-25.25	<2.2e-16
O13-19	A18-256	-24.74	<2.2e-16
O13-19	A19-1	-24.38	<2.2e-16
O13-19	Q135.2	-24.19	<2.2e-16
A18-256	A19-1	-24.67	<2.2e-16
A18-256	Q135.2	-25.30	<2.2e-16
A19-1	Q135.2	-25.02	<2.2e-16

Table S7 Accession numbers of strains that have pCP13-like family plasmids .

Strain	Plasmid	Accession No.
13	pCP13	AP003515.1
NY83906550	pCPNY83906550-1	MK285071.1
T1	pCPT1	MK285059.1
JXJA17	p1	NZ_MK275619.1

Table S8-1 Virulence genes included in this study.

Toxins and enzymes	Gene name	Accession No.
BECa	*becA*	NC_023918.1
BECb	*becB*	NC_023918.1
Phospholipase C	*plc*	D63911.1
CPE	*cpe*	M98037.1
Collagenase	*colA*	D13791.1
Beta-toxin	*cpb*	KP064410.1
Beta-2 toxin	*cpb2 variant 1*	AP003515.1
Beta-2 toxin	*cpb2 variant 2*	CP009558.1
Perfringolysin O	*pfoA*	BA000016.3
Epsilon-toxin	*etx*	M95206.1
Iota-toxin component Ia	*iap*	NC_015712.1
Iota-toxin component Ib	*ibp*	NC_015712.1
NetB	*netB*	FJ189503.1
Peptidase	*tpeL*	EU848493.1
NetF	*netF*	KJ606986
Lambda-toxin	*lam*	AJ439340
Alveolysin	*alv*	TGY44713.1

Table S8-2 Antimicrobial genes included in this study.

Antibiotic resistance product	Gene name	Accession No.
TetM	*tetM*	CYYX01000001.1
BcrA	*bcrA*	GU810181.1
BcrB	*bcrB*	GU810180.1
BcrD	*bcrD*	GU810179.2
BcrR	*bcrR*	GU810182.1
chloramphenicol resistance protein	*crp*	NZ_CABPRN010000011.1
